# A scoping review on the community dividend resulting from testing and treating hepatitis C infection in people living in detention

**DOI:** 10.1017/S0950268824001419

**Published:** 2024-12-05

**Authors:** Eszter Kiss-Farina, Chizoba Esio-Bassey, Emma Plugge, Nick De Viggiani

**Affiliations:** 1 UK Health Security Agency, South Colonnade, UK; 2 University of the West of England, Bristol, UK; 3 University of Southampton, Southampton, UK

**Keywords:** hepatitis C, infectious disease epidemiology, prevalence of disease, estimating, public health, screening programme, prison health

## Abstract

A scoping review was conducted to map out sources, types, characteristics of evidence that substantiate the existence of a community dividend arising from testing and treating hepatitis C virus (HCV) infection in people living in detention – where community dividend is defined as the benefit of prison-related intervention for general population health. Joanna Briggs Institute methodology guidance was used. Literature search was done in EMBASE, Scopus, ASSIA, UWE library, CINAHL Plus, and Medline to find studies published in any country, any language between January 1991 and June 2022. PRISMA ScR flow chart mapped out the number of records identified, included, and reasons for exclusion. Data were extracted and charted in Excel. The findings were systematically reported by charting table headings then synthesized in the discussion. Quality assessment was carried out. The descriptive analysis demonstrated economic, clinical, and epidemiological domains to the community dividend in long-term health expenditure savings, reduction in HCV-related disease sequelae, increase in survival, improvement in quality of life, and reduction in infection transmission, most of which are realized in the community following release. Therefore, targeting marginalized populations affected by HCV could expedite the elimination effort, reduce inequalities, and have a positive impact on the wider population.

## Introduction

Seventy-one million people live with hepatitis C virus (HCV) worldwide [1]. Globally, HCV disproportionately affects incarcerated individuals. People living in detention (PLD) are 9–13 times more likely to be HCV infected than the general population [2]. Of the estimated 10.2 million PLD worldwide on any given day in 2013 [3], Dolan *et al.* estimated that more than 1.5 million (15.1%) were living with HCV [4].

Since 2014, highly effective, well-tolerated direct-acting antivirals (DAAs) with shorter treatment duration have allowed for the completion of treatment within the average length of imprisonment, which is 8.5 months globally [5, 6]. As the disease can have a long asymptomatic phase, early detection and treatment can prevent spread, progression, and reduce long-term healthcare costs.

PLD are from the poorest and most marginalized sections of the population [7]. They often serve multiple, short-term sentences [5] due to crimes of poverty – violations committed primarily out of economic necessity – and drug-related offences – which are a leading causes of imprisonment globally, particularly in countries with punitive drug policies [8]. PLD spend most of their lives outside of prison, so treating them while they are inside reduces onward transmission risk in the community. Therefore, prison-related interventions such as the micro-elimination of HCV in places of detention by universal screening and treatment of the infection will not only deliver benefits to the individual but are likely to create a community dividend, that is, benefit for general population health [9]. Such interventions that focus on equivalent health outcomes for PLD and the general population – rather than equivalent healthcare – are more likely to successfully contribute to equity in prison health [10]. They may also impact on community-level health disparities by providing healthcare to PLD that might not have been accessible prior or following their incarceration, thus helping to reduce health disparities when these individuals return to their communities.

The potential positive effect of prison-related HCV interventions on prison populations and the wider community has been highlighted numerous times but no one has yet synthesized the evidence that examines whether and in what ways HCV diagnosis and treatment of PLD benefit general population health. Therefore, a scoping review was conducted with the aim of mapping out sources, types, and characteristics of evidence on the existence of a community dividend and to identify key outcomes that make up the community dividend.

## Inclusion criteria

### Participants

Population of interest was defined as people living in detention or in secure psychiatric units for any length of time of all ages – including young offenders – and of all genders and sexualities. Recently released PLD or those on parole who were tested in prison, started treatment there or awaiting to start treatment in the community were also included. We considered sources reporting on both whole general prison populations and prison population sub-groups (e.g., injecting drug users living in detention, HIV-infected incarcerated individuals, etc.).

We excluded people living in police custody, at immigration removal centres, prisoners of war, and individuals recently released from detention or on parole if they were not offered an HCV test or/and treatment while in detention.

### Concept

We included evidence to support or refute a community dividend caused by testing and/or treating HCV in detention. Community dividend was defined as the benefit of a prison-related intervention for general population health [11]. We excluded evidence with no clear link to a community dividend caused by testing and/or treating HCV in detention. Evidence was excluded when the HCV intervention resulting in the presence or absence of a community dividend was other than testing and/or treatment such as programmes directed at awareness raising, behaviour change, provision of syringes for IDUs, opioid substitution therapy, and so forth.

### Context

The context was HCV infection. The markers and manifestations that made evidence eligible for inclusion were the following: HCV infection, HCV-induced liver disease, extrahepatic manifestations associated with HCV, mental and physical illnesses that are the consequences of HCV infection, health statuses, and levels of wellbeing that are associated with the presence and absence of HCV infection.

Markers that were not associated with HCV infection were excluded unless they could not be separated from HCV-associated outcomes.

### Types of sources

We included multiple evidence sources and study types to allow for a broad conceptualization of the community dividend.

We included:

- primary and secondary research, health economic evaluations directed at testing and/or treating HCV in prison population where at least testing took place while already in custody,
- modelling studies whose outcome was a community dividend (e.g., transmission prevention, disease progression prevention, opportunity cost, etc.),
- and all other literature published or unpublished that discussed the community dividend resulting from testing and/or treating HCV in detained populations.

We excluded secondary research unless they contained novel information beyond the primary sources they reviewed; thus, excluded systematic reviews without meta-analysis.

## Methods

The Joanna Briggs Institute (JBI) scoping review methodology guidance [12, 13] was followed. The literature was searched from January 1991 to June 2022 because it was assumed that research activity directed at secure settings might have become more prominent after the publishing of the United Nations’ Basic Principles for the Treatment of Prisoners in 1991 [14]. No limitation was set on the country of origin and language.

Database searches were carried out in EMBASE (Table 1), Scopus, ASSIA, UWE Library, CINAHL Plus, and MEDLINE using the keywords in the logic grid displayed in Table 2. The searches were conducted in titles and abstracts without limits except for the limitation of publication period. Search results were exported into Zotero, duplicates were removed manually, and the remaining items were screened by reading the titles and abstracts. Of the 63 records sought for retrieval, 21 could be included and are listed in Table 3: 19 published articles [15–33] and two conference posters [34–35]. The reference lists of included literature were searched as per JBI protocol. While more information could not be retrieved on the content of the two conference posters, they were included for more comprehensive mapping of the geography and type of evidence available on the topic.

**Table 1.** Example library search

Search in EMBASE on 1 June 2022–3404 results
1. (“hepatitis C” or “hep C” or HCV or “blood borne” or bloodborne or BBV or “liver ADJ4 disease” or “liver ADJ4 fibrosis” or “liver ADJ4 cirrhosis” or “viral hepatitis”).mp. [mp=title, abstract, heading word, drug trade name, original title, device manufacturer, drug manufacturer, device trade name, keyword heading word, floating subheading word, candidate term word]
2. limit 1 to yr= “1991 –Current”
3. (prison* or incarcerat* or inmate* or detain* or jail* or detention or offend* or custod* or remand* or correcti* or criminal* or penitentiar* or imprison* or “penal institut*”).mp. [mp=title, abstract, heading word, drug trade name, original title, device manufacturer, drug manufacturer, device trade name, keyword heading word, floating subheading word, candidate term word]
4. limit 3 to yr= “1991 –Current”
5. (intervention* or screen* or test* or treat* or therap* or “case-finding” or “case finding” or daa* or “direct-acting antiviral*” or “direct acting antiviral*” or medic*).mp. [mp=title, abstract, heading word, drug trade name, original title, device manufacturer, drug manufacturer, device trade name, keyword heading word, floating subheading word, candidate term word]
6. limit 5 to yr= “1991 –Current”
7. 2 and 4 and 6

**Table 2.** Logic grid

Population	Intervention			Outcome measure
prison* incarcerat* inmate* detain* jail* detention offend* custod* remand* correcti* criminal* penitentiar* imprison* penal institut*	hepatitis C* Hep C HCV “bloodborne” bloodborne BBV “liver fibrosis” “liver disease*” “liver cirrhosis” “viral hepatitis”	AND	intervention* screen* test* treat* therap* case-finding “case finding” DAA* “direct-acting antiviral*” “direct acting antiviral*”	benefit* impact* association* dividend* outcome* econom* societ* effectiv* cost-effectiv* “cost effectiv*” saving save* prevent* community public “general population”

**Table 3.** Reference list of included sources

1	**Palmer A, *et al*.** (2021) A costing analysis of a state-wide, nurse-led hepatitis C treatment model in prison. *International Journal of Drug Policy.* **94**: 103203. [15]
2	**Marco A, Dominguez-Hernandez R., and Casado MA.** (2020) Cost-effectiveness analysis of chronic hepatitis C treatment in the prison population in Spain. *Revista espanola de sanidad penitenciaria.* **22**: 66–74. [16]
3	**Assoumou SA, *et al.*** (2020) Cost-effectiveness and budgetary impact of hepatitis C virus testing, treatment, and linkage to care in US prisons. *Clinical Infectious Diseases.* **70**: 1388–1396. [17]
4	**Martin NK, et al.** (2013) Cost-effectiveness of increasing HCV case-finding for people who inject drugs via dried blood spot testing in addiction services and prisons. *Journal of Hepatology.* **58**: 403–404. [18]
5	**Ward Z, *et al.*** (2021) Cost-effectiveness of mass screening for Hepatitis C virus among all inmates in an Irish prison. *International Journal of Drug Policy.* **96**: 103394. [19]
6	**Mohamed Z, *et al.*** (2020) Cost-effectiveness of strategies to improve HCV screening, linkage-to-care and treatment in remand prison settings in England. *Liver International.* **40:** 2950–2960. [20]
7	**Sutton AJ, Edmunds WJ, Gill ON.** (2006) Estimating the cost-effectiveness of detecting cases of chronic hepatitis C infection on reception into prison. *BMC Public Health.* **6**: 170. [21]
8	**Chen C-P, *et al.*** (2019) Evaluation of cost-effectiveness of peginterferon plus ribavirin for chronic hepatitis C treatment and direct-acting antiviral agents among HIV-infected patients in the prison and community settings. *Journal of Microbiology, Immunology and Infection.* **4:** 556–562. [22]
9	**Kwon JA, *et al.*** (2021) Hepatitis C treatment strategies in prisons: A cost-effectiveness analysis. *PLoS ONE.* **16**: e0245896. [23]
10	**Nicolas Perez D, *et al*.** (2022) Hepatitis C virus infection screening reduces mortality and is cost-effective independently of the intervention test. *Revista espanola de enfermedades digestivas : organo oficial de la Sociedad Espanola de Patologia Digestiva.* **114**: 731–737. [24]
11	**Chhatwal J, *et al***. (2018) Improved health outcomes from hepatitis C treatment scale-up in Spain’s prisons: A cost-effectiveness study. *Journal of Hepatology.53rd Annual Meeting of the European Association for the Study of the Liver, International Liver Congress 2018. Paris France.* **68**: S151. [25]
12	**Martin NK, *et al***. (2016) Is increased hepatitis C virus case-finding combined with current or 8-week to 12-week direct-acting antiviral therapy cost-effective in UK prisons? A prevention benefit analysis. *Hepatology.* **63:** 1796–1808. [26]
13	**Girardin F, *et al***. (2019) Modelling the Impact and Cost-effectiveness of Extended Hepatitis C Virus Screening and Treatment with Direct-acting Antivirals in a Swiss Custodial Setting. *Clinical Infectious Diseases.* **69**: 1980–1986. [27]
14	**Stone J, *et al.*** (2017) Modelling the impact of incarceration and prison-based hepatitis C virus (HCV) treatment on HCV transmission among people who inject drugs in Scotland. *Addiction.* **112:** 1302–1314. [28]
15	**He T, *et al*.** (2016) Prevention of hepatitis C by screening and treatment in U.S. prisons. *Annals of Internal Medicine.* **164**: 84–92. [29]
16	**Liu S, *et al*.** (2014) Sofosbuvir-Based Treatment Regimens for Chronic, Genotype 1 Hepatitis C Virus Infection in U.S. Incarcerated Populations. *Annals of Internal Medicine.* **161**: 546–553. [30]
17	**Sutton AJ, *et al.*** (2008) The cost-effectiveness of screening and treatment for hepatitis C in prisons in England and Wales: A cost-utility analysis. *Journal of Viral Hepatitis.* **15:** 797–808. [31]
18	**Castelnuovo E, *et al.*** (2006) The cost-effectiveness of testing for hepatitis C in former injecting drug users. *Health Technology Assessment.* [online]. **10:** 32. [32]
19	**Godin A, *et al.*** (2021) The role of prison-based interventions for hepatitis C virus (HCV) micro-elimination among people who inject drugs in Montreal, Canada. *International Journal of Drug Policy.* **88:** 102738. [33]
20	**Wong JB, *et al***. (2013) Cost-effectiveness of hepatitis c treatment by primary care providers supported by the Extension for Community Healthcare Outcomes (ECHO) Model. *Hepatology.64th Annual Meeting of the American Association for the Study of Liver Diseases: The Liver Meeting 2013. Washington, DC United States.* **58:** 330A. [34]
21	**Manca F, *et al.*** (2020) HCV screening strategies targeting prisoners and immigrants from endemic countries: are they cost-effective? *Journal of Hepatology.EASL: The Digital International Liver Congress.* **73**: S806. [35]

**Figure 1.**
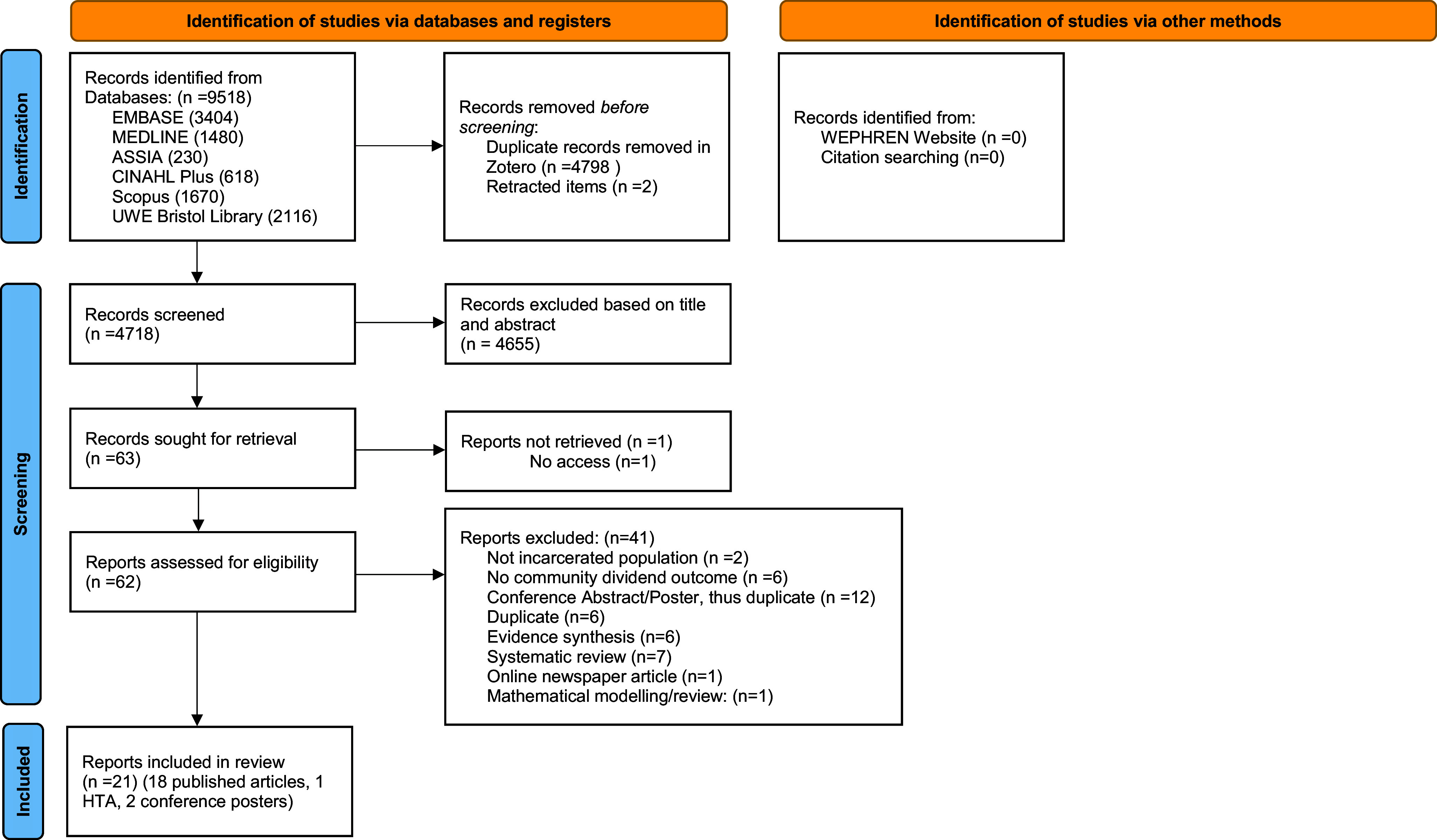
PRISMA Flow diagram *From:* Page MJ, McKenzie JE, Bossuyt PM, Boutron I, Hoffmann TC, Mulrow CD, et al. The PRISMA 2020 statement: an updated guideline for reporting systematic reviews. BMJ 2021;372:n71. doi: 10.1136/bmj.n71. For more information, visit: http://www.prisma-statement.org/

PRISMA ScR flow diagram was completed (Figure 1). It depicts the flow of information through the different phases of the scoping review study selection and maps out the number of records identified, included, and excluded as well as the reasons for exclusion.

Relevant data that relate to the review question and objectives were extracted and charted (Table 4) using the following refined charting table headings: Study Number, Reference, Title, Year, Country, Study Type, Aim(s), Methodology, Population, Description of Intervention(s) and comparator (covers Test (T) and/or Treatment (Tx) and/or Linkage to Care (LtC)), Outcome Measures, Key Findings, Domain of Community Dividend. For each included source of evidence, findings were collated and reported by charting table headings, thus providing an overview of the characteristics of the sources of evidence, the distribution of studies by year and country of publication, the distribution of study designs, intervention types, target populations, and methodologies used. Using the completed charting table, a list of outcomes relating to the community dividend was collated and their distribution across the included sources illustrated.

**Table 4.** Charting table

Studies (N=21)	[15]	[16]	[17]	[18]	[19]	[20]	[21]	[22]	[23]	[24]	[25]
Reference	(Palmer A. *et al.*, 2021)	(Marco A., Dominguez-Hernandez R., and Casado M.A., 2020)	(Assoumou S.A. *et al.*, 2020)	(Martin N.K. *et al.*, 2013)	(Ward Z. *et al.*, 2021)	(Mohamed Z. *et al.*, 2020)	(Sutton A.J., Edmunds W.J., and Gill O.N., 2006)	(Chen C.-P. *et al.*, 2019)	(Kwon J.A. *et al.*, 2021)	(Nicolas Perez D. *et al.*, 2022)	(Chhatwal J. *et al.*, 2018)
Title	A costing analysis of a state-wide, nurse-led hepatitis C treatment model in prison	Cost-effectiveness analysis of chronic hepatitis C treatment in the prison population in Spain	Cost-effectiveness and budgetary impact of hepatitis C virus testing, treatment, and linkage to care in US prisons	Cost-effectiveness of HCV case-finding for people who inject drugs via dried blood spot testing in specialist addiction services and prisons	Cost-effectiveness of mass screening for Hepatitis C virus among all inmates in an Irish prison	Cost-effectiveness of strategies to improve HCV screening, linkage-to-care and treatment in remand prison settings in England	Estimating the cost-effectiveness of detecting cases of chronic hepatitis C infection on reception into prison	Evaluation of cost-effectiveness of peginterferon plus ribavirin for chronic hepatitis C treatment and direct-acting antiviral agents among HIV-infected patients in the prison and community settings	Hepatitis C treatment strategies in prisons: A cost-effectiveness analysis	Hepatitis C virus infection screening reduces mortality and is cost-effective independently of the intervention test	Improved health outcomes from hepatitis C treatment scale-up in Spain’s prisons: A cost-effectiveness study
Year	2021	2020	2020	2013	2021	2020	2006	2019	2021	2022	2018
Country	Australia	Spain	US	UK	Ireland	England	England and Wales	Taiwan	Australia	Spain	Spain
Study Type	Costing analysis	CEA (CUA) Modelling	CEA and Budgetary Impact (BI) modelling	CEA-CUA modelling	CEA modelling	CEA modelling	CEA modelling	CEA based on observational studies	CEA modelling	CEA modelling	CEA and Budgetary Impact (BI) modelling
Methodology	Ingredients-based costing approach to costing analysis	CEA using lifetime Markov model to simulate treatment and disease progression	CEA and BI modelling using individual-level transition simulation model of HCV testing, treatment, and linkage to care	CUA using a dynamic, deterministic model of incarceration, HCV transmission, disease progression and HCV treatment	CEA using a dynamic HCV transmission and disease progression model among incarcerated and community PWID calibrated to the Dublin HCV epidemic	CEA using de novo closed-cohort decision tree and Markov state transition model to simulate accrued costs and health-related outcomes of HCV testing and treatment	CEA using Markov decision analysis model embedded in a model of the flow of IDUs through prison	CEA of PegIFN/RBV and DAA therapy using SVR and cost per treatment data of current study and two other observational studies	CEA using dynamic mathematical model of HCV transmission accounting for key risk behaviours, prison dynamics, natural history of HCV-related liver disease, healthcare costs	CEA using Markov model	CEA using agent-based simulation Markov model includes dynamic movement of people in and out of prison, transmission between PWID, natural history of HCV, treatment with DAAs, awareness of status
Aim(s)	Estimate the average non-drug cost of initiating a prisoner on treatment using a nurse-led approach and compare it with the cost of primary and hospital-based models of care in the community	Evaluate cost-effectiveness of DAA treatment versus no treatment in prison dwellers, analyse the clinical and economic impact on liver complications and mortality	Estimate clinical outcomes, cost-effectiveness, budgetary impact of HCV testing and treatment in US prisons or linkage to care at release	Determine the cost-effectiveness of increasing HCV case-finding among PWID by offering DBS testing in specialist addiction services and prisons	Evaluate the cost-effectiveness of mass HCV screening intervention and linkage to care	Assess cost-effectiveness of traditional and simplified screening and treatment in a remand prison	Estimates the average cumulative cost of identifying a new case of HCV in prison and the cost effectiveness of alternative HCV case-finding scenarios	survey the SVR and cost-effectiveness of PegIFN/RBV treatment in prisons and in the community among HIV-infected and non HIV-infected patients with different genotypes then compare cost-effectiveness between PegIFN/RBV and DAAs among HIV-infected patients stratified by genotype	Assess the impact of increasing DAA treatment uptake on HCV incidence and prevalence in NSW and the cost-effectiveness of alternate treatment strategies, to estimate the lifetime burden of disease, costs and changes in QALYs in prison and in the community	Compare the cost-effectiveness of 3 screening methods for HCV (HCV-Ab serology, DBS HCV-Ab, HCV RNA by DBS) in different settings according to low (general population) and high prevalence (prisons and addiction centres)	Identify a cost-effective strategy to scale up HCV treatment in all Spain’s prisons and project the long-term clinical and economic benefits
Description of intervention(s) and comparator (covers Test (T) and/or Treatment (Tx) and/or Linkage to Care (LtC))	T and Tx initiation under state-wide prison hepatitis program (SHP) versus hospital-based and prison-based care in the community (T-Tx (initiation only))	CHC prison dwellers treated with DAAs over 2 yrs versus no treatment (T-Tx)	15 strategies/permutations of testing (risk factor based, routine entry or at release), treatment (fibrosis 3 or above, all), and linkage to care (at release) versus no test-no treat-no linkage to care (T-Tx-LtC)	DBS (3.6 fold increase in addiction services and 2.6 in prisons) versus baseline current venepuncture testing with or without LtC (T-Tx-LtC)	HepCheck intervention = mass screening in one prison (419 screened, 12 treated) and scaling up mass screening to all 5 prisons yearly and every 3 yrs versus standard-of-care (SOC) of intermittent screening on committal (20 treatments per year) (T-Tx-LtC)	Six scenarios compared the varied rates of screening (47–90%), linkage to care (60–86%) and treatment uptake (21–85%) that characterise the status quo national average, universal general prison population screening, high-risk prison population using DBS screening, high-uptake DBS screening or a simplified pathway (T-Tx)	Five case-finding scenarios: permutations of verbal screen or no screen for ever having received past positive HCV test and/or injected illicit drugs versus do nothing (no verbal screening, no testing) (T)	Comparisons of cost-effectiveness between PegIFN/RBV and DAAs used among HIV-infected patients stratified by HCV genotype (Tx)	Four models reflecting different average prison length of stay (LOS) of 2, 6, 24, 36 mths, each model considered 4 DAA coverage scenarios (status quo 10%, 25%, 50%, 90%) (T-Tx)	Three screening strategies (HCV-Ab serology, DBS HCV-Ab, HCV RNA by DBS) in low risk/prevalence and high risk/prevalence populations with 50% and 100% participation versus no screening (T-Tx)	Four strategies compared with status quo, 1 – eligible >6 mths LOS prioritize by fibrosis stage treatment capacity 200/yr irrespective of region, 2 – eligible >6mths LOS prioritize prisons by their prevalence with treatment capacity of 200/yr irrespective of fibrosis stage, 3 – eligible >6 mths LOS unlimited capacity, 4 – everyone eligible unlimited capacity (Tx)
Outcome measures	Average non-drug cost per individual of initiating HCV treatment	QALYs gained, cost-effectiveness expressed in terms of ICUR (cost per QALY), reduction in cases of liver complications and HCV-related mortality	Impact on liver fibrosis, LC, life expectancy, proportion (%) of lifetime SVR, healthcare cost prison entrant, QALYs gained, cost-effectiveness expressed in terms of ICER (cost per QALY)	Cost effectiveness expressed in terms of ICER (cost per QALY)	QALYs gained, cost-effectiveness expressed in terms of ICER (cost per QALY), net monetary benefit (NMB) calculated of intervention scenarios, impact on prevalence and incidence	QALYs gained, cost effectiveness in terms of ICER (cost per QALY), impact on liver disease burden and mortality	Cost-effectiveness expressed in terms of ICER (cumulative discounted cost per chronic HCV (CHC) detected)	Cost-effectiveness expressed in terms of ICER (cost per SVR achieved)	Incidence rate and prevalence in 2045, cumulative liver-related deaths (2015–2045), QALYs gained, cost-effectiveness expressed in terms of ICER (cost per QALY), NMB	QALYs gained, cost-effectiveness expressed in terms of ICERs (cost per QALY), mortality due to LC, HCC, LT	projected HCV-related deaths, DC, HCC until 2050, HCV transmission and death which resulted from inmates whose HCV was not treated in prison, total population-level QALYs gained, cost-effectiveness in terms of ICER (cost per QALY), BI
Key findings	The average cost per treatment initiation in prison was cheaper than both hospital-based and primary-based care in the community	Treating prison dwellers is a cost-effective strategy, treatment of all CHC achieved an additional 5 QALYs compared to no treatment, ICUR €690 per QALY well below the WTP threshold (€21,000–30,000) used in Spain, avoided cases of DC (92%), HCC (83%), LT (90%), LrD (88%)	Most extensive strategies substantially reduced liver fibrosis and lifetime cumulative prevalence of LC, increased the proportion of lifetime SVR, cost-effectiveness: T all-Tx all-no LtC additional 0.1374 discounted QALY, ICER $19,000/QALY and, T all-Tx all-LtC ICER $24,000/QALY, expanding T had little impact unless Tx or LtC follows, testing only at release and risk-based testing are inefficient allocation of resources, restricting to liver fibre 3 or greater was also dominated, with lower DAA price also treating lower fibrosis state becomes non-dominated, 1 yr BI: appealing ICER but unaffordable to many prisons (89% of pharmacy budget)	Case-finding by DBS not cost-effective (£59,400 per QALY), introducing continuity of care increases cost-effectiveness, ICER bellow WTP threshold (£20,000) when continuity of care >40%, in the base case, most PWID treatment initiated in prison were interrupted	Mass screening in Mountjoy Prison was cost-effective gained a mean additional 3.8 QALYs over 50yrs, an ICER of €9,552/QALY, scaling up to all prisons is also cost-effective regardless of yearly or 3 yearly screening, but yearly has a greater NMB (€7,393,382), Under SOC, 56.0% decrease in chronic HCV prevalence and 55.9% in incidence in Dublin over 2017–2030. The intervention has little additional impact on these projections because of its low coverage, with a median of 1.0 disease-related death and 6.3 infections averted over 50 years due to the 12 additional individuals treated	All strategy ICERs fell under the national WTP threshold, optimising cascade of care is cost-effective, where universal screening is not practical stratified approach focused on intensive screening and treatment of PWID which resulted in increased uptake and highly cost-effective. 70% remained in mild disease state (19% status quo), less pronounced differences in advanced liver disease between groups. DC lower, HCC equivalent, mortality lower among treated individuals. 1.12–1.24 QALYs gained due to intervention.	Administering verbal screening for a past positive HCV test and for ever having engaged in illicit drug use prior to test have an impact on the cost-effectiveness, the least cost-effective is identifying only those who have not received a test yet, also less cost-effective when no verbal screening (as far too many test are done), all scenarios become less cost-effective as time passes due to IDUs returning to prison who are aware of their infection, for all: cost increases up to a plateau then remains constant	Receiving treatment in the community was unfavourable factor of SVR and incurred higher cost per SVR achieved. HIV coinfection, baseline viral load, lack of RVR did not have impact on SVR rates. In terms of cost-effectiveness, DAAs may be more suitable for HCV G1 and G6 because the cost per SVR was similar to those achieved with PegINF/RBV regardless of the medical cost of DAAS which was the highest at the time of the study.	Sharp decline in new HCV infections in all LOS scenarios (9%–65%). Prevalence showed a linear increase over time, regardless of the average LOS, and lower prevalence with higher treatment coverage. Number of cumulative LrD (2015–2045) was lower with longer LOS and more intensive treatment. Across settings and all LOS, DAA was highly cost-effective (highest ICER under $600 well below the WTP threshold ($28,000), 1.36–3.23 QALYs gained across strategies). This is confirmed by the positive NMB at all levels of coverage (economic surplus $6–10,000 per treated prisoner at 25%, over $40,000 at 90%).	DBS-anti-HCV was the most cost-effective strategy in the population with high (50%) prevalence (PWID, prisons), for 50% participation, the ICERs for all three screening methods versus no screening were between €1,816–2,151 well below the corresponding low population ICERs of €12,015-€13,633, highest gain in QALYs 14.75. Participation below 30% made the screening strategies inefficient, dominated by no screening. The mortality reduction for screening versus no screening was between 24.1% and 80% depending on strategy.	Status quo prevalence marginal decrease (14.4–>11%), St1–4 substantial reduction to 2.7–3.7% by 2030, substantial reduction in DC, HCC, LRD, and incidence. Among LrD prevented by scaling up 88–90% could have occurred in the community,98–99% of the HCV transmission averted would have occurred in the community, CEA: strategies 1–3 dominated by strategy 4 (ICER €9,602 per QALY, BI: annual HCV-associated cost in status quo 12M, St 1–4 96.2–406.5M but by 2030 decrease to 43.2M
Domain of community dividend	Economic	Economic – clinical	Economic – clinical	Economic	Economic-clinical-epidemiological	Economic – clinical	Economic	Economic	Economic – clinical – epidemiological	Economic – clinical	Economic – clinical – epidemiological

Studies (N=21)	[26]	[27]	[28]	[29]	[30]	[31]	[32]	[33]	[34]	[35]
Reference	(Martin N.K. *et al.*, 2016)	(Girardin F. *et al.*, 2019)	(Stone J. *et al.*, 2017)	(He T. *et al.*, 2016)	(Liu S. *et al.*, 2014)	(Sutton A.J. *et al.*, 2008)	(Castelnuovo E. *et al.*, 2006)	(Godin A. *et al.*, 2021)	(Wong J.B. *et al.*, 2013)	(Manca F. *et al*, 2020)
Title	Is increased hepatitis C virus case-finding combined with current or 8-week to 12-week direct-acting antiviral therapy cost-effective in UK prisons? A prevention benefit analysis	Modelling the Impact and Cost-effectiveness of Extended Hepatitis C Virus Screening and Treatment with Direct-Acting Antivirals in a Swiss Custodial Setting	Modelling the impact of incarceration and prison-based hepatitis C virus (HCV) treatment on HCV transmission among people who inject drugs in Scotland	Prevention of hepatitis C by screening and treatment in U.S. prisons	Sofosbuvir-based Treatment Regimens for Chronic, Genotype 1 Hepatitis C Virus Infection in U.S. Incarcerated Populations	The cost-effectiveness of screening and treatment for hepatitis C in prisons in England and Wales: A cost-utility analysis	The cost-effectiveness of testing for hepatitis C in former injecting drug users	The role of prison-based interventions for hepatitis C virus (HCV) micro-elimination among people who inject drugs in Montreal, Canada	Cost-effectiveness of hepatitis c treatment by primary care providers supported by the Extension for Community Healthcare Outcomes (ECHO) Model	HCV screening strategies targeting prisoners and immigrants from endemic countries: are they cost-effective?
Year	2016	2019	2017	2016	2014	2008	2006	2021	2013	2020
Country	UK	Switzerland	Scotland	US	US	England and Wales	UK	Canada	US	Scotland
Study Type	CEA modelling	CEA modelling	epidemiological modelling	CEA & Budget Effect modelling	CEA modelling	CUA modelling	Health Technology Assessment	epidemiological modelling	poster on CEA modelling	poster on CEA modelling
Methodology	CEA using dynamic model that tracks incarceration, injecting behaviour, HCV transmission among PWID, testing and treatment through various settings	CEA that combines a five-year de novo decision tree screening model with results from a Markov model of HCV treatment outcomes	dynamic deterministic mathematical modelling of incarceration among PWID using approximate Bayesian sequential Monte Carlo scheme computation for model calibration	CEA using agent-based microsimulation model (TapHCV – treatment as prevention of HCV) of disease and its progression both in prisons and the general population	CEA using decision analytic Markov model to follow cohorts (stratified by liver fibrosis stage, genotype, race, age), allow reinfection and treatment initiation after release	cost-utility analysis that considers the costs and benefits of testing and treatment of two hypothetical cohorts	CUA-CEA using decision tree and Markov models to investigate the impact of testing, diagnosis and treatment on progression of HCV in a hypothetical cohort	dynamic compartmental deterministic mathematical model of HCV transmission among PWID that considers the dynamics of HCV transmission, incarceration and injecting behaviours, Bayesian framework for model calibration	used a previously published and validated cost-effectiveness model to compare ECHO-facilitated HCV Tx to no antiviral therapy	two high risk populations were identified: prisoners and high prevalence ethnic minorities. CEA of HCV screening strategies using Markov model was undertaken for each population
Aim(s)	Assess the cost-effectiveness of increased HCV testing and treatment rates in English prisons	Explore the cost-effectiveness of increased HCV screening to cover all PLD	Estimate the contribution of incarceration to the Scottish HCV epidemic among PWID – ‘population-attributable fraction’ PAF, estimate the 15-year impact of existing and future prison-based prevention and HCV treatment interventions on HCV incidence and chronic prevalence among PWID	Evaluate the health and economic effect of HCV screening and treatment in prisons on the HCV epidemic in society, understand the complex dynamics between prison-related interventions and disease burden in society as a whole	Assess the cost-effectiveness of Sofosbuvir for HCV treatment in incarcerated population	investigate the cost-utility of administering a single round of screening on reception into prison to all individuals	explore the effectiveness and cost-effectiveness as well as the impact on disease progression of case finding and treatment at various settings	examine the effect of prison-based test-and-treat and test and post-release linkage to care strategies on HCV transmission, and the added benefits of post-release risk-reduction measures, DAA scale up in prison and in the community	examine the cost-effectiveness of ECHO for HCV, comparing antiviral treatment costs, travel and lost work time costs to ECHO personnel costs (no disease costs)	evaluate the cost-effectiveness of a range of strategies for diagnosing HCV in specific high-risk populations in Scotland (UK)
Description of intervention(s) and comparator (covers Test (T) and/or Treatment (Tx) and/or Linkage to Care (LtC))	Three strategies, 1 – doubling of testing and current tx, 2 – doubling of testing and 8–12 wk IFN free DAA in prison + current tx in community, 3 – tx as in 1 and 2 but tx scale-up for PWID – up to 25% tx after referral versus HCV voluntary risk-based screening – 6% of prison entrants, current tx (T-Tx)	Comprehensive screening strategy of PLD versus current approach – risk-based screening strategy (screening PLD who indicate they are from high-risk population) (T-Tx)	Potential future prison-associated prevention and HCV treatment interventions (80% tx scale up, post release risk prevention, no incarceration of PWID) versus existing prevention and HCV treatment interventions (Tx)	Five scenarios: no screening, 1yrRisk1 (1-time risk-based screening of currently incarcerated and entrants with active/former IDU for 1 year – assumed uptake 75%), 1yrAll (1-time opt-out universal screening of currently incarcerated and entrants for 1 year – assumed uptake 90%), 5yrsAll, 10yrsAll, in the general population combination of birth-cohort and risk-based screening versus current standard treatment (T-Tx)	No treatment, two-drug therapy (pegylated interferon and ribavirin), or 3-drug therapy with either boceprevir or sofosbuvir. For inmates with short remaining sentences (<1.5 years), only no treatment or sofosbuvir three-drug therapy were feasible; for those with long sentences (≥1.5 years; mean, 10 years), all strategies were considered. After release, eligible persons could receive sofosbuvir three-drug therapy (Tx)	Testing (eligible who responds in the positive to questions regarding IDU) and treatment is offered on reception into prison followed by the possibility of spontaneous presentation for screening and possible treatment in a community location versus only presentation for screening and possible treatment in a community may occur (T-Tx)	Case-finding and treatment in prison, general practice and drug services (and subgroup analyses based on duration of infection) versus no case-finding but spontaneous presentation for testing (T-Tx)	1 – prison-based test and treat (PB T 90%-Tx 75%) 2 – prison-based test (90%) and post-release linkage to care (PB T-LtC Tx 75% in community), 3 – PB T-Tx + R and 4 – PB T-Tx + R (with complimentary measures that reduce heightened post-release risk of HCV by 50%) versus 1- T-Tx and R (risk reduction intervention) at 2018 levels, 2 – community-based DAAs scale-up (T-Tx-LtC)	ECHO compared to no antiviral therapy (Tx)	Novel screening strategies such as opt-out testing policies in prisons and community outreach activities offering dry blood spot testing versus standard care diagnostic pathways (T)
Outcome measures	Total QALYs gained due to intervention, cost-effectiveness in terms of ICER (cost per QALY), changes in chronic prevalence and incidence	cost-effectiveness expressed as ICER (cost per QALY), cost of screening per HCV-positive person linked to tx, NMB	population-attributable fraction’ (PAF) of incarceration to HCV transmission among PWID, decrease in HCV incidence and chronic prevalence due to existing OST, HCV treatment, potential decriminalisation of drug use, new interventions that prevent elevated risk of transmission after release, scaling up DAAs to 80%	number of new HCV cases resulting from untreated positives being released, budgetary impact on prison system, reduction in HCV disease cost, total QALYs gained in prison and in community due to intervention, cost-effectiveness expressed as ICER (cost per QALY and cost per CHC detected), reduction in cases of DC, HCC, LT, LrD	QALYs gained, cost-effectiveness expressed in terms of ICER (cost per QALY), SVRs, reductions in DC, HCC, LT	QALYs gained, cost-effectiveness expressed in terms of ICER (cost per QALY)	cases of DC, HCC, LT, LrD prevented, ICER (cost per LY), QALYS gained, ICUR (cost per QALY)	impact on chronic HCV prevalence, incidence, the cumulative fraction of first new chronic infections prevented among people without prior exposure to HCV over 2018–2030, prevention of new cases per person treated in prison	QALYs gained, cost-effectiveness expressed in terms of ICER (cost per QALY)	A decision tree explored the incremental cost per additional positive patient detected, and a Markov model was employed to present incremental cost per QALYs gained and NMB
Key findings	Doubling testing with status quo tx is borderline cost-effective: 421.27 QALYs gained, ICER £19,851, with DAAs 171.25 QALYS gained, ICER £15,090 likely to be cost-effective, increased tx for PWID to 10% (ICER £12,691 current tx, £6461 DAA)and 25% (ICER £8000 current tx, £4000 DAAs), changes in chronic prevalence and incidence – baseline negligible impact <1%, doubled testing and current tx 1% over 50 years, doubled testing and DAAs + 25% tx scale up 12% relative reduction, the only modest epidemiological impact is due to low effective tx rates for PWID given the gaps in the cascade of care	comprehensive test is cost-effective compared to current screening with base-case ICER CHF 14,312 (WTP 100,000), NMB for whole target population CHF 23,298, CHF 4,298 per person, total incremental proportion of PLD tested increased: 13.6%–>67%, proportion of HCV-pos population diagnosed increased: 35.4%–>63.7%, cost of screening per HCV pos person linked to treatment: CHF 13,942 in comprehensive and CHF 5,011 in current	Incarceration contributes nearly a 1/3 of all current HCV transmission among PWID, with current rates of HCV DAA treatment incidence decreases 10.7% and prevalence 9.7%, if no prison treatment it would still decrease due to community treatment 10% 8.9%, without current prison OST decrease 3.1% 4.7%, preventing future incarceration of PWID + current treatment reduction 21.9% 16.9%, current tx and prison OST with intervention preventing heightened risk among recently released PWID decrease 45% 33.3%, prison tx scaled up to 80% (>16 wk LOS) decrease 45.6% 45.5%, if heightened transmission risk is also prevented with 80% scale up in prison decrease 70.2% 66.5%, if 80% (>12 wks LOS) 55.8% 55.9%, with risk prevention post release 76.4% 74.4%	**Cost-effectiveness expressed as** cost per CHC detected $880-$2030 across the scenarios, ICERs $19,600-$29,200 (cost per additional QALY), benefit on society exclusively by excluding QALYs gained inside prison: increase in QALYs 35,600–90,300 – corresponding ICERs $23,100–35,400, reduction in HCV disease cost: by 260–760M of which 82–84% of saving would have been caused by prison screening, budget effect on prison system: first-year cost under risk-based $900M, under opt-out$1,146M, 12,4% over the current health budget of state and federal prisons in the US, however, over 15 years, only and additional 0.7% of the current healthcare budget, number of new infections prevented when compared to no-screening scenario 5,500–12,700, number of LRD 4,200–11,700 – 80% of these would have occurred in the outside community, LT 300–900, HCC 3,000–8,600, DC 2,600–7,300	The strategies yielded 13.12, 13.57, 14.43, and 15.18 QALYs, respectively, for persons with long sentences. Sofosbuvir produced the largest absolute reductions in DC (16%) and HCC (9%), resulting in 2.1 additional QALYs. For persons with short sentences, sofosbuvir cost $25 700 per QALY gained compared with no treatment; for those with long sentences, it dominated other treatments, costing $28 800 per QALY gained compared with no treatment, Sofosbuvir three-drug therapy highly effective compared to alternatives, higher SVR rates produced clinical benefits: reduction in DC, life-expectancy gain of 1.6 yrs compared to no treatment.	93 individuals treated as a result of case-finding as opposed to 500 subsequently presented in community locations as a result of raised awareness, the incremental benefit of screening is small 0.005 QALY/patient, the cost/QALY gained is £54,852 well above the WTP threshold, 15–24 yrs of age is the most-cost-effective scenario (£40,227) with smallest budget impact, the value of prison screening comes from raising awareness, reducing case-finding cost, increasing treatment acceptance and adherence would ensure more cost-effectiveness (considers T-Tx)	case-finding prevents 3 cases of DC, 3 LrDs, 1 case of HCC (at 30 years), 25 additional people are likely to undergo combination therapy per 1000 people approached, 1 LT prevented for 10,000 people approached, systematically offering testing to 1000 people result in 1 LY gained for an investment of £20,084, 0.046 QALY gained, ICUR £16,514 per QALY, cost-effectiveness of case-finding in different settings is similar, although the absolute cost and benefits vary considerably, 2 approaches to case-finding in prison were considered based on studies in Dartmoor and the Isle of Wight prisons and analysis on data from Dartmoor had the least favourable cost-effectiveness of the strategies considered (£20,000 per QALY), subgroup analysis showed case-finding is the most cost-effective in people whose infection is more long-standing (at greater risk of progression – case-finding yields £15,000 per QALY	over 2018–2030, **status quo** slight decreases in prevalence (4%) and incidence (3%), **T-Tx scale up in community** substantial decreases 88% and 84%, prevent 1.2 new first chronic infection per person treated over 18–30, **PB T-Tx without T-Tx scale up in community** relative incidence reduction of 48% compared to 2018 and prevent a cumulative 22% of new first chronic infections, **PB T-L without T-Tx scale up in community** incidence reduction of 36% and cumulative 15% new first chronic infections, **PB T-Tx + R and PB T-L + R without T-Tx scale up in community** incidence reduction 54% and 44%, **PB T-Tx with T-Tx scale up in community** incidence decreased 96% and 30% of new first chronic infections were prevented and every additional person treated in prison averted 23 new first chronic infections, the same **with treatment in community (L)** 94% 22% 13, **together with (R)** Risk reduction intervention – incidence reduction 97% 95% and prevented up to a third of new chronic infections	ECHO access to HCV treatment increased discounted quality-adjusted life expectancy by 3.8 years, 3.5 in the community and 4.2 in the prison dwellers. Lower lifetime costs and higher QALYs for 62% of the 261 patients and 55% of the community and 70% of the prison dwellers. Among the non-dominated patients, the ICER of ECHO averaged $8300 per QALY, $9400 in the community and $5900 in prison dwellers, well below the WTP threshold. When comparing only antiviral treatment costs and travel and lost work time costs to ECHO costs (no disease costs), the mean savings from ECHO were $1352 per person or >$350,000 for the 261 patients. For 10% of patients, travel costs were lower than ECHO costs because of their geographic proximity to the academic centre.	Screening prisoners for HCV with opt-out testing resulted in a 7.82-fold increase in detecting positive individuals compared to screening based on symptomatic detection, costing £366 per any additional detected individual. In the lifetime analysis, both the strategies are highly cost-effective. ICER £1,195per QALY for the prison population.
Domain of community dividend	Economic-clinical-epidemiological	Economic	Epidemiological	Economic – clinical – epidemiological	Economic – clinical	Economic-clinical	Economic – clinical	Epidemiological	Economic-clinical	Economic-clinical

As per JBI guidance, the formal assessment of the methodological quality of the included studies was not performed with the aim of providing a basis for inclusion or to facilitate the development of recommendations for practice. It was carried out to determine the trustworthiness and rigour of the included sources, to confirm the quality of the evidence base, and to help draw well-founded dependable conclusions. The Consolidated Health Economic Evaluation Reporting Standards (CHEERS) criteria list [36], an appropriate tool for the quality assessment of both economic and non-economic modelling studies, was used.

## Results

Most of the included studies were published after 2014 except for 5 [18, 21, 31–32, 34] published between 1991 and 2013. The studies of the scoping review were sourced from a very limited number of high-income countries – United Kingdom [18, 20–21, 26, 28, 31–32, 35], United States [17, 29–30, 34], Spain [16, 24–25], Australia [15, 23], Canada [33], Ireland [19], Switzerland [27], and Taiwan [22].

The aim of the included papers was to evaluate the impact of an HCV intervention or interventions on either the individual living in detention, the prison community, the wider community, or the combination of these. Some evaluated only the economic [15, 18, 21–22, 27], some only the epidemiological impact [28, 33]; others a combination of economic, clinical, and epidemiological impacts: economic and clinical [16–17, 20, 24, 30–32, 34–35], economic, clinical, and epidemiological [19, 23, 25–26, 29].

The interventions could be grouped into four distinctive types according to their coverage of the HCV care cascade: two sources evaluated the impact of HCV testing strategies only [21, 35], five HCV treatment strategies only [22, 25, 28, 30, 34], and ten studies evaluated both testing and treatment [15–16, 20, 23–24, 26–27, 29, 31–32]. Four sources also discussed the effect of linkage to care (LtC) in the community in addition to testing and treatment [17–19, 33] and demonstrated the positive impact on economic, clinical, and epidemiological markers.

19 of the 21 included studies [16–21, 23–35] used prospective longitudinal modelling – economic or epidemiological – as their research method to simulate the long-term costs and/or individual and population benefits of different HCV cascade of care strategies and scenarios. Among these, there were 11 cost-effectiveness analysis (CEA) modelling studies [16, 18–21, 23–24, 26–27, 30–31], two posters on CEA modelling studies [34–35], one health technology assessment/CEA modelling study [32], three CEA and budgetary impact modelling studies [17, 25, 29], and two epidemiological modelling studies [28, 33]. Two studies did not use modelling. One of them was a CEA [22] based on retrospective observational cohort studies. Finally, the second non-modelling study [15] had an ingredients-based costing approach to costing analysis.

Table 5 provides a visual representation grid of the community dividend-related outcomes and their distribution across the studies. Charting the data facilitated the collation of outcomes with 20 eventual items. Each item related to the community dividend and could be placed into three distinctive groups depending on the impact of the intervention: in the economic, the clinical, and/or the epidemiological domain(s). The green cells on the grid show where a positive outcome was demonstrated, the red cell where the community dividend of the outcome was refuted.

**Table 5.** Community dividend-related outcomes and their distribution across the included sources

Domains	Community dividend-related outcomes	[15]	[16]	[17]	[18]	[19]	[20]	[21]	[22]	[23]	[24]	[25]	[26]	[27]	[28]	[29]	[30]	[31]	[32]	[33]	[34]	[35]
Economic	Cost-effectiveness – ICER (cost per chronic HCV CHC) detected																					
	Cost of screening per HCV-positive person linked to treatment																					
	Cost-effectiveness – ICER (cost per QALY)																					
	Cost-effectiveness – ICER (cost per SVR achieved)																					
	Reduction in HCV disease cost																					
	Cost of treatment initiation																					
	Net monetary benefit																					
	Budgetary impact																					
Clinical	Reduction in fibrosis (cirrhosis)																					
	Reduction in lifetime cumulative prevalence of LC																					
	Reduction in DC and HCC																					
	Reduction in cases of DC, HCC, LT																					
	Reduction in HCV-related deaths																					
	Increase in proportion of lifetime SVR																					
	QALYs gained																					
Epidemiological	Reduction in prevalence																					
	Reduction in incidence																					
	Prevention of cumulative % new first chronic infections																					
	Prevention of new cases per person treated in prison																					
	Number of new HCV cases resulting from untreated positives being released																					

ICER – incremental cost-effectiveness ratio, CHC – chronic hepatitis (CHC), QALY – Quality-Adjusted Life-Year, SVR – sustained virologic response, LC – liver cirrhosis, DC – decompensated cirrhosis, HCC – hepatocellular carcinoma, LT – liver transplant

Both individual-level benefits of prevention of disease progression and population-level benefits of prevention of HCV transmission were demonstrated in the included literature. The cost-saving outcomes that provided an economic rationale for implementing more intensive prison-based HCV testing and treatment were the reduction in HCV disease cost [29], the favourable cost of treatment initiation in the prison setting [15] as opposed to community settings, and the positive net monetary benefit [19, 23, 27, 35]. Budget impact analyses in the three studies [17, 25, 29] provided an invaluable argument against budget holders’ reluctance to invest in correctional health. The positive economic impact was demonstrated by 17 of the 18 studies [16–27, 29–30, 32, 34–35] that completed an economic evaluation by measuring the cost-effectiveness of interventions from the healthcare provider’s or societal perspective. They found that the opportunity costs did not exceed the benefits of the interventions when the benefit was expressed as quality-adjusted life-years (QALYs) gained [16–20, 23–27, 29–30, 32, 34–35], sustained virologic response (SVR) achieved [22], chronic hepatitis (CHC) detected [21, 29, 35], or the cost of screening per HCV-positive person linked to treatment [27]. Cost-effectiveness was refuted in one study that was published in 2008 [31].

The clinical benefit was measured across the studies by the changes recorded in the number of cases in specific liver disease stages. One third of the sources provided evidence of the reduction in one or more of the following: fibrosis, decompensated cirrhosis, hepatocellular carcinoma, liver transplant, and liver-related death [16–17, 24–25, 29–30, 32]. These were lifetime cumulative measurements or projections for at least 30 years, which meant that the positive effects of testing and treating PLD well exceeded the length of their prison stay and would have impacted mostly on the community. Another outcome is the increase in the proportion of lifetime SVR [17]. Fourteen CEAs gave a value to the health benefit of their interventions in QALYs [16–17, 19–20, 23–26, 29–32, 34–35]. They measured how many additional years of life a PLD and people in the community may gain due to different HCV test and/or treatment scenarios by being cured and/or by not getting infected.

The most reported outcomes in the epidemiological domain were the changes in HCV prevalence [19, 23, 25–26, 28, 33] and incidence [19, 25–26, 28, 33] computed for the overall population, not just the prison population. Reduction was found in point prevalence in all the studies that measured the proportion of the population with HCV at a specific future time [23, 25–26, 28]. Studies reporting on cumulative prevalence or period prevalence [19, 33] – quantifying the change in the HCV-infected proportion of the population over a period of time – also described reduction. Reduction was seen in incidence due to interventions described by five studies [19, 25–26, 28, 33]. They modelled the difference in the number of new HCV cases in a population over a given interval of time (1 year) attributed to interventions and their comparators. The prevention effect on the prevalence and incidence of different testing (T) – treatment (Tx) – linkage to care (LtC) strategies – varying in intensity and their coverage of the cascade of care – was compared in a study [33] by the cumulative percentages of new first chronic infections that could be prevented between 2018 and 2030. The prevention of new cases per person treated in prison for the different scenarios also captured the community dividend [33]. The same prevention benefit was expressed in a converse way in one source [29] where the number of new HCV cases resulting from untreated positives being released into the community was given as an epidemiological outcome.

The main author and a health economist completed the quality assessment of the included sources using the CHEERS criteria list [36] (Table 6). Overall, 11 studies were assessed as good quality, one study was of moderate quality and 6 studies were assessed as high quality. Almost all the studies identified their studies as an economic evaluation except for two studies [29–30]. Only one study [32] indicated whether a health economic plan was developed. All the studies except two [20–21] provided characteristics of the study population, including age range, demographics, socioeconomic, and clinical characteristics. All the studies provided details of alternative interventions or strategies compared. All studies stated the perspective adopted and why it was chosen except one [22]. Most of the studies stated the time horizon adopted and why it was appropriate except two studies [22, 27]. Only a few studies [16, 18, 25–27, 29–32] described how costs were valued. All the studies described methods to characterize any sources of uncertainty in the study. All included studies reported mean values of costs and outcomes except one study [24], effects of uncertainty from analytic judgements and input parameters on findings. All studies reported key findings and limitations of study.

**Table 6.** Quality assessment using CHEERS

Section/topic	Item No	Guidance for reporting	[15] (Palmer A. *et al.*, 2021)	[16] (Marco A., Dominguez-Hernandez R., and Casado M.A., 2020)	[17] (Assoumou S.A. *et al.*, 2020)	[18] (Martin N.K. *et al.*, 2013)	[19] (Ward Z. *et al.*, 2021)	[20] (Mohamed Z. *et al.*, 2020)	[21](Sutton A.J., Edmunds W.J., and Gill O.N., 2006)	[22] (Chen C.-P. *et al.*, 2019)	[23] (Kwon J.A. *et al.*, 2021)	[24] (Nicolas Perez D. *et al.*, 2022)	[25] (Chhatwal J. *et al.*, 2018)	[26] (Martin N.K. *et al.*, 2016)	[27] (Girardin F. *et al.*, 2019)	[28] (Stone J. *et al.*, 2017)	[29] (He T. *et al.*, 2016)	[30] (Liu S. *et al.*, 2014)	[31] (Sutton A.J. *et al.*, 2008)	[32] (Castelnuovo E. *et al.*, 2006)	[33] (Godin A. *et al.*, 2021)
Title																					
Title	1	Identify the study as an economic evaluation and specify the interventions being compared.	✓	✓	✓	✓	✓	✓	✓	✓	✓	✓	✓	✓	✓	N/A	✗	✗	✓	✓	N/A
Abstract																					
Abstract	2	Provide a structured summary that highlights context, key methods, results, and alternative analyses.	✓	✓	✓	✓	✓	✓	✓	✓	✓	✓	✓	✓	✓	✓	✓	✓	✓	✓	✓
Introduction																					
Background and objectives	3	Give the context for the study, the study question, and its practical relevance for decision making in policy or practice.	✓	✓	✓	✓	✓	✓	✓	✓	✓	✓	✓	✓	✓	✓	✓	✓	✓	✓	✓
Methods																					
Health economic analysis plan	4	Indicate whether a health economic analysis plan was developed and where available.	✗	✗	✗	✗	✗	✗	✗	✗	✗	✗	✗	✗	✗	N/A	✗	✗	✗	✓	N/A
Study population	5	Describe characteristics of the study population (such as age range, demographics, socioeconomic, or clinical characteristics).	✗	✓	✓	✓	✓	✗	✗	✓	✓	✓	✓	✓	✓	✓	✓	✓	✓	✓	✓
Setting and location	6	Provide relevant contextual information that may influence findings.	✗	✓	✗	✓	✗	✓	✓	✗	✓	✓	✓	✓	✓	✓	✓	✓	✓	✓	✓
Comparators	7	Describe the interventions or strategies being compared and why chosen.	✓	✓	✓	✓	✓	✓	✓	✓	✓	✓	✓	✓	✓	✓	✓	✓	✓	✓	✓
Perspective	8	State the perspective(s) adopted by the study and why chosen.	✓	✓	✓	✓	✓	✓	✓	✗	✓	✓	✓	✓	✓	N/A	✓	✓	✓	✓	N/A
Time horizon	9	State the time horizon for the study and why appropriate.	✓	✓	✓	✓	✓	✓	✓	✗	✓	✓	✓	✓	✗	✓	✓	✓	✓	✓	✓
Discount rate	10	Report the discount rate(s) and reason chosen.	✓	✓	✓	✓	✓	✓	✓	✗	✓	✓	✗	✓	✓	N/A	✓	✓	✓	✓	N/A
Selection of outcomes	11	Describe what outcomes were used as the measure(s) of benefit(s) and harm(s).	✓	✓	✓	✓	✓	✓	✓	✓	✓	✓	✓	✓	✓	✓	✓	✓	✓	✓	✓
Measurement of outcomes	12	Describe how outcomes used to capture benefit(s) and harm(s) were measured.	✓	✓	✗	✗	✓	✓	✓	✓	✓	✓	✓	✓	✓	✓	✓	✓	✓	✓	✓
Valuation of outcomes	13	Describe the population and methods used to measure and value outcomes.	✓	✓	✗	✗	✓	✓	✓	✓	✓	✓	✓	✓	✓	✓	✓	✓	✓	✓	✓
Measurement and valuation of resources and costs	14	Describe how costs were valued.	✓	✓	✗	✓	✗	✗	✗	✗	✗	✗	✓	✓	✓	N/A	✓	✓	✓	✓	N/A
Currency, price date, and conversion	15	Report the dates of the estimated resource quantities and unit costs, plus the currency and year of conversion.	✓	✓	✓	✓	✓	✗	✓	✗	✗	✗	✓	✓	✓	N/A	✓	✓	✓	✓	N/A
Rationale and description of model	16	If modelling is used, describe in detail and why used. Report if the model is publicly available and where it can be accessed.	N/A	✓	✓	✓	✓	✓	✓	N/A	✓	✓	✓	✓	✓	✓	✓	✓	✓	✓	✓
Analytics and assumptions	17	Describe any methods for analysing or statistically transforming data, any extrapolation methods, and approaches for validating any model used.	✓	✓	✗	✗	✓	✗	✗	✗	✗	✗	✓	✓	✗	✓	✓	✗	✗	✓	✓
Characterizing heterogeneity	18	Describe any methods used for estimating how the results of the study vary for subgroups.	✗	✗	✓	✓	✓	✗	✓	✓	✓	✓	✓	✓	✗	✓	✓	✓	✗	✓	✗
Characterizing distributional effects	19	Describe how impacts are distributed across different individuals or adjustments made to reflect priority populations.	✓	✗	✓	✓	✗	✗	✗	✓	✗	✓	✓	✓	✗	✗	✓	✓	✓	✓	✗
Characterizing uncertainty	20	Describe methods to characterize any sources of uncertainty in the analysis.	✓	✓	✓	✓	✓	✓	✓	✓	✓	✓	✓	✓	✓	✓	✓	✓	✓	✓	✓
Approach to engagement with patients and others affected by the study	21	Describe any approaches to engage patients or service recipients, the general public, communities, or stakeholders (such as clinicians or payers) in the design of the study.	✓	✗	✗	✗	✗	✗	✗	✗	✗	✗	✗	✗	✗	✗	✗	✗	✗	✗	✗
Results																					
Study parameters	22	Report all analytic inputs (such as values, ranges, references) including uncertainty or distributional assumptions.	✓	✗	✓	✓	✓	✓	✓	✓	✓	✗	✓	✓	✓	✓	✓	✓	✓	✓	✓
Summary of main results	23	Report the mean values for the main categories of costs and outcomes of interest and summarize them in the most appropriate overall measure.	✓	✓	✓	✓	✓	✓	✓	✓	✓	✗	✓	✓	✓	✓	✓	✓	✓	✓	✓
Effects of uncertainty	24	Describe how uncertainty about analytic judgments, inputs, or projections affect findings. Report the effect of choice of discount rate and time horizon, if applicable.	✓	✓	✓	✓	✓	✓	✓	N/A	✓	✓	✓	✓	✓	✓	✓	✓	✓	✓	✓
Effect of engagement with patients and others affected by the study	25	Report on any difference patient/service recipient, general public, community, or stakeholder involvement made to the approach or findings of the study	✓	✗	✗	✗	✗	✗	✗	✗	✗	✗	✗	✗	✗	✗	✗	✗	✗	✗	✗
Discussion																					
Study findings, limitations, generalizability, and current knowledge	26	Report key findings, limitations, ethical or equity considerations not captured, and how these could affect patients, policy, or practice.	✓	✓	✓	✓	✓	✓	✓	✓	✓	✓	✓	✓	✓	✓	✓	✓	✓	✓	✓
Other relevant information																					
Source of funding	27	Describe how the study was funded and any role of the funder in the identification, design, conduct, and reporting of the analysis	✓	✓	✓	✓	✓	✗	✓	✗	✓	✓	✓	✓	✓	✓	✓	✓	✓	✓	✓
Conflicts of interest	28	Report authors conflicts of interest according to journal or International Committee of Medical Journal Editors requirements.	✓	✓	✓	✓	✓	✓	✓	✓	✓	✓	✓	✓	✓	✓	✓	✓	✗	✓	✓
Total score			22/27	22/28	21/28	22/28	22/28	18/28	21/28	17/26	21/28	20/28	24/28	25/28	21/28	17/22	24/28	23/28	22/28	26/28	18/22
Criteria met (%)			81%	79%	75%	79%	79%	64%	75%	65%	75%	71%	86%	89%	75%	77%	86%	82%	79%	93%	82%
Quality			high	good	good	good	good	moderate	good	good	good	good	high	high	good	good	high	high	good	high	high

Score	Quality																				
80% and above	High																				
65% and above	Good																				
50% and above	Moderate																				
35% and above	Poor																				
20% and above	Very poor																				

## Discussion

HCV is an important global health issue, and it is particularly significant for people in places of detention, as health inequalities disproportionately affect them [2]. Several studies have shown the effect on the general population of HCV testing and treatment interventions carried out in places of detention by focusing on some of the measurable outcomes. These studies have all been included in our review. Our study is the first to introduce the concept of community dividend to the research and stakeholder community, synthesising and describing all the outcomes that are related to it.

Our results demonstrate that the community dividend of testing for HCV in places of detention and treating chronic HCV-infected incarcerated individuals comprise 20 measurable outcomes within three major domains: economic, clinical, and epidemiological. It is a cost-effective public health strategy and increasingly so with the recent availability of DAAs. Case-finding and treatment are a good investment of taxpayers’ money and result in savings in long-term health expenditure. The cost is amply compensated by individual and collective benefits. Cost-effectiveness was refuted by only one study [31]. However, the study took place in 2008 before the availability of well-tolerated DAAs that have a shorter treatment duration, and the researchers themselves noted that improved treatment acceptance and adherence would ensure more cost-effectiveness.

All studies evidenced that testing and treating HCV in PLD reduces the incidence of HCV-related liver complications, increases survival, improves quality of life for both the prison population and the general population, and reduces infection transmission. Most of the benefits are realized in the community following release.

Our scoping review provides invaluable evidence that can significantly contribute to evidence-based policymaking and the design of interventions aimed at scaling up HCV testing and treatment in detention facilities. For a healthcare intervention to be thoroughly appraised and recommended for practice, evidence must encompass not only effectiveness but also appropriateness and feasibility [37]. We have identified major gaps in the existing evidence base as only one aspect of feasibility—budgetary impact—was covered in the studies reviewed, and none of the included sources considered appropriateness. These gaps could be addressed by various research methods, for example, observational, interpretive, and descriptive studies, focus groups, action research, case studies, expert opinion, and so forth. As for improving the evidence of effectiveness, taking a broader perspective when carrying out future economic research could present HCV interventions as more cost-effective. Cost-consequence analysis, preferably as a supplement to CEA would allow consideration of non-health-related or difficult to quantify outcomes such as equity. Social return on investment analysis would be able to account for broader value and outcomes, social, economic, and environmental benefits [38].

The main limitation of this scoping review is that the main search, study selection, and data charting and collating results were conducted by only the main author as part of their master’s dissertation project although co-authors viewed a sample of included studies. Bias was minimized by adherence to JBI guidance, completion of PRISMA ScR Checklist (Table 7) and frequent meetings and discussions.

**Table 7.** Preferred Reporting Items for Systematic reviews and Meta-Analyses extension for Scoping Reviews (PRISMA-ScR) Checklist

SECTION	ITEM	PRISMA-ScR CHECKLIST ITEM	REPORTED ON PAGE #
**TITLE**			
Title	1	Identify the report as a scoping review.	✓
**ABSTRACT**			
Structured summary	2	Provide a structured summary that includes (as applicable): background, objectives, eligibility criteria, sources of evidence, charting methods, results, and conclusions that relate to the review questions and objectives.	✓
**INTRODUCTION**			
Rationale	3	Describe the rationale for the review in the context of what is already known. Explain why the review questions/objectives lend themselves to a scoping review approach.	✓
Objectives	4	Provide an explicit statement of the questions and objectives being addressed with reference to their key elements (e.g., population or participants, concepts, and context) or other relevant key elements used to conceptualize the review questions and/or objectives.	✓
**METHODS**			
Protocol and registration	5	Indicate whether a review protocol exists; state if and where it can be accessed (e.g., a Web address); and if available, provide registration information, including the registration number.	✓
Eligibility criteria	6	Specify characteristics of the sources of evidence used as eligibility criteria (e.g., years considered, language, and publication status), and provide a rationale.	✓
Information sources*	7	Describe all information sources in the search (e.g., databases with dates of coverage and contact with authors to identify additional sources), as well as the date the most recent search was executed.	✓
Search	8	Present the full electronic search strategy for at least one database, including any limits used, such that it could be repeated.	✓
Selection of sources of evidence†	9	State the process for selecting sources of evidence (i.e., screening and eligibility) included in the scoping review.	✓
Data charting process‡	10	Describe the methods of charting data from the included sources of evidence (e.g., calibrated forms or forms that have been tested by the team before their use, and whether data charting was done independently or in duplicate) and any processes for obtaining and confirming data from investigators.	✓
Data items	11	List and define all variables for which data were sought and any assumptions and simplifications made.	✓
Critical appraisal of individual sources of evidence§	12	If done, provide a rationale for conducting a critical appraisal of included sources of evidence; describe the methods used and how this information was used in any data synthesis (if appropriate).	✓
Synthesis of results	13	Describe the methods of handling and summarizing the data that were charted.	✓
**RESULTS**			
Selection of sources of evidence	14	Give numbers of sources of evidence screened, assessed for eligibility, and included in the review, with reasons for exclusions at each stage, ideally using a flow diagram.	✓
Characteristics of sources of evidence	15	For each source of evidence, present characteristics for which data were charted and provide the citations.	✓
Critical appraisal within sources of evidence	16	If done, present data on critical appraisal of included sources of evidence (see item 12).	✓
Results of individual sources of evidence	17	For each included source of evidence, present the relevant data that were charted that relate to the review questions and objectives.	✓
Synthesis of results	18	Summarize and/or present the charting results as they relate to the review questions and objectives.	✓
**DISCUSSION**			
Summary of evidence	19	Summarize the main results (including an overview of concepts, themes, and types of evidence available), link to the review questions and objectives, and consider the relevance to key groups.	✓
Limitations	20	Discuss the limitations of the scoping review process.	✓
Conclusions	21	Provide a general interpretation of the results with respect to the review questions and objectives, as well as potential implications and/or next steps.	✓
**FUNDING**			
Funding	22	Describe sources of funding for the included sources of evidence, as well as sources of funding for the scoping review. Describe the role of the funders of the scoping review.	N/A

JBI = Joanna Briggs Institute; PRISMA-ScR = Preferred Reporting Items for Systematic reviews and Meta-Analyses extension for Scoping Reviews.

* Where *sources of evidence* (see second footnote) are compiled from, such as bibliographic databases, social media platforms, and Web sites.

† A more inclusive/heterogeneous term used to account for the different types of evidence or data sources (e.g., quantitative and/or qualitative research, expert opinion, and policy documents) that may be eligible in a scoping review as opposed to only studies. This is not to be confused with *information sources* (see first footnote).

‡ *The frameworks by Arksey and O’Malley (6) and Levac and colleagues (7) and the JBI guidance (4, 5) refer to the process of data extraction in a scoping review as data charting.*

§ The process of systematically examining research evidence to assess its validity, results, and relevance before using it to inform a decision. This term is used for items 12 and 19 instead of “risk of bias” (which is more applicable to systematic reviews of interventions) to include and acknowledge the various sources of evidence that may be used in a scoping review (e.g., quantitative and/or qualitative research, expert opinion, and policy document).

Tricco et al. [41].

Although we aimed to include a wide range of sources, the current review can be characterized by homogeneity in study types, mainly providing evidence of effectiveness via economic evaluations. The validity and trustworthiness of the evidence however is supported by the high-ranking research methods of the included sources as well as the quality assessment of the included papers, which was good overall. The quality assessment was performed by two independent reviewers, the main author and a health economist, to reduce bias.

Another limitation of the findings is the geographical spread of the located studies. Most sources were from the United Kingdom and the United States and a few other high-income countries (Spain, Australia, Taiwan, Canada, Switzerland, Ireland). Middle- and low-income countries were not represented at all. This has been previously documented in prison research [39]. Investment in research and elimination programmes remains low in many parts of the world due to lack of political commitment and domestic and international financing [40]. It must be noted that some of the factors influencing the community dividend cannot be separated from characteristics of local political systems, economies, and epidemics such as the degree of penalization of drug use, average length of prison stay (which might determine eligibility), drug availability, the efficiency of drugs used, prevalence and incidence of HCV infection and of intravenous drug use, and other HCV risk factors in prisons and in the community. Therefore, we expect that research aimed at providing evidence of effectiveness, appropriateness, and feasibility would vary geographically.

The authors have demonstrated that HCV testing and/or treatment interventions in PLD greatly benefit general population health by resulting in long-term health expenditure savings, reduction in HCV-related disease sequalae, increase in survival, improvement in quality of life, reduction in infection transmission, and most of these benefits are seen in the community. Understanding the considerable impact that testing and/or treating HCV in PLD have on general population health will inform stakeholder decisions to invest in testing and treatment services for PLD, which will be crucial for achieving HCV elimination and for reducing inequalities both within and outside the prison wall.

## Data Availability

All sources that support my findings are included in the reference list and can be accessed individually. All data extracted from the 21 sources that met inclusion criteria for the scoping review can be found in the attached Excel table entitled charting table.

## References

[1] World Health Organization (2017) Global hepatitis report 2017. Available at: https://www.who.int/publications/i/item/9789241565455. (accessed 25 October 2023).

[2] Larney S, et al. (2013) Incidence and prevalence of hepatitis C in prisons and other closed settings: results of a systematic review and meta-analysis. Hepatology. 58: 1215–1224. 23504650 10.1002/hep.26387 PMC3723697

[3] Walmsley R. (2013) World prison population list (tenth addition). London: International Centre for Prison Studies. Available at: http://www.prisonstudies.org/sites/default/files/resources/downloads/wppl_10.Pdf. (accessed 25 October 2023).

[4] Dolan K, et al. (2016) Global burden of HIV, viral hepatitis, and tuberculosis in prisoners and detainees. The Lancet. 388: 1089–1102. 10.1016/S0140-6736(16)30466-4 27427453

[5] Aebi MF, Tiago MM. (2020) *SPACE 1 Council of Europe annual penal statistics 2020.* Available at: https://wp.unil.ch/space/files/2021/04/210330_FinalReport_SPACE_I_2020.pdf. (accessed 25 October 2023).

[6] Ermis F, Senocak Tasci E. (2015) New treatment strategies for hepatitis C infection. World Journal of Hepatology. 7: 2100–2109. 26301052 10.4254/wjh.v7.i17.2100 PMC4539403

[7] Karstedt S. (2021) Inequality and punishment: a global paradox? Journal of Criminology. 54: 5–20.

[8] Harm Reduction International (2022) Global State of Harm Reduction 2022. Available at: https://hri.global/wp-content/uploads/2022/11/HRI_GSHR-2022_Full-Report_Final.pdf. (accessed 26 January 2024).

[9] Stürup-Toft S, O‘Moore EJ, Plugge EH. (2018) Looking behind the bars: emerging health issues for people in prison. British Medical Bulletin. 125: 15–23. 29394343 10.1093/bmb/ldx052

[10] Charles A, Draper H. (2012) ‘Equivalence of care’ in prison medicine: is equivalence of process the right measure of equity? Journal of Medical Ethics. 38: 215–218. 21955956 10.1136/medethics-2011-100083

[11] O’Moore É. (2015) The community dividend: why improving prisoner health is essential for public health. Available at: https://ukhsa.blog.gov.uk/2015/07/06/the-community-dividend-why-improving-prisoner-health-is-essential-for-public-health/. (accessed 25 October 2023).

[12] Peters MDJ, et al. (2015) Guidance for conducting systematic scoping reviews. International Journal of Evidence-Based Healthcare. 13: 141–146. 26134548 10.1097/XEB.0000000000000050

[13] Peters MDJ, et al. (2020) Updated methodological guidance for the conduct of scoping reviews. JBI Evidence Synthesis. 18: 2119–2126. 33038124 10.11124/JBIES-20-00167

[14] United Nations General Assembly (UNGA) (1991) Basic principles for the treatment of prisoners, resolution 45/111. Available at: https://www.ohchr.org/sites/default/files/basicprinciples.pdf. (accessed 26 January 2024).

[15] Palmer A, et al. (2021) A costing analysis of a state-wide, nurse-led hepatitis C treatment model in prison. International Journal of Drug Policy. 94: 103203. 33744667 10.1016/j.drugpo.2021.103203

[16] Marco A, Dominguez-Hernandez R., and Casado MA. (2020) Cost-effectiveness analysis of chronic hepatitis C treatment in the prison population in Spain. Revista espanola de sanidad penitenciaria. 22: 66–74. 32697276 10.18176/resp.00012 PMC7537362

[17] Assoumou SA, et al. (2020) Cost-effectiveness and budgetary impact of hepatitis C virus testing, treatment, and linkage to care in US prisons. Clinical Infectious Diseases. 70: 1388–1396. 31095676 10.1093/cid/ciz383 PMC7318776

[18] Martin NK, et al. (2013) Cost-effectiveness of increasing HCV case-finding for people who inject drugs via dried blood spot testing in addiction services and prisons. Journal of Hepatology. 58: 403–404. 23943776 10.1136/bmjopen-2013-003153 PMC3752052

[19] Ward Z, et al. (2021) Cost-effectiveness of mass screening for Hepatitis C virus among all inmates in an Irish prison. International Journal of Drug Policy. 96: 103394. 34412938 10.1016/j.drugpo.2021.103394 PMC9179078

[20] Mohamed Z, et al. (2020) Cost-effectiveness of strategies to improve HCV screening, linkage-to-care and treatment in remand prison settings in England. Liver International. 40: 2950–2960. 32750192 10.1111/liv.14628

[21] Sutton AJ, Edmunds WJ, Gill ON. (2006) Estimating the cost-effectiveness of detecting cases of chronic hepatitis C infection on reception into prison. BMC Public Health. 6: 170. 16803622 10.1186/1471-2458-6-170 PMC1543636

[22] Chen C-P, et al. (2019) Evaluation of cost-effectiveness of peginterferon plus ribavirin for chronic hepatitis C treatment and direct-acting antiviral agents among HIV-infected patients in the prison and community settings. Journal of Microbiology, Immunology and Infection. 4: 556–562. 10.1016/j.jmii.2018.10.002 30360951

[23] Kwon JA, et al. (2021) Hepatitis C treatment strategies in prisons: a cost-effectiveness analysis. PLoS ONE. 16: e0245896. 33571196 10.1371/journal.pone.0245896 PMC7877645

[24] Nicolas Perez D, et al. (2022) Hepatitis C virus infection screening reduces mortality and is cost-effective independently of the intervention test. Revista espanola de enfermedades digestivas: organo oficial de la Sociedad Espanola de Patologia Digestiva. 114: 731–737. 10.17235/reed.2022.8609/2022 35285662

[25] Chhatwal J, et al. (2018) Improved health outcomes from hepatitis C treatment scale-up in Spain’s prisons: a cost-effectiveness study. Journal of Hepatology. 53rd Annual Meeting of the European Association for the Study of the Liver, International Liver Congress 2018. Paris France. 68: S151.

[26] Martin NK, et al. (2016) Is increased hepatitis C virus case-finding combined with current or 8-week to 12-week direct-acting antiviral therapy cost-effective in UK prisons? A prevention benefit analysis. Hepatology. 63: 1796–1808. 26864802 10.1002/hep.28497 PMC4920048

[27] Girardin F, et al. (2019) Modelling the Impact and Cost-effectiveness of Extended Hepatitis C Virus Screening and Treatment with Direct-acting Antivirals in a Swiss Custodial Setting. Clinical Infectious Diseases. 69: 1980–1986. 30715266 10.1093/cid/ciz088

[28] Stone J, et al. (2017) Modelling the impact of incarceration and prison-based hepatitis C virus (HCV) treatment on HCV transmission among people who inject drugs in Scotland. Addiction. 112: 1302–1314. 28257600 10.1111/add.13783 PMC5461206

[29] He T, et al. (2016) Prevention of hepatitis C by screening and treatment in U.S. prisons. Annals of Internal Medicine. 164: 84–92. 26595252 10.7326/M15-0617 PMC4854298

[30] Liu S, et al. (2014) Sofosbuvir-based treatment regimens for chronic, genotype 1 hepatitis C virus infection in U.S. Incarcerated Populations. Annals of Internal Medicine. 161: 546–553. 25329202 10.7326/M14-0602 PMC4313741

[31] Sutton AJ, et al. (2008) The cost-effectiveness of screening and treatment for hepatitis C in prisons in England and Wales: a cost-utility analysis. Journal of Viral Hepatitis. 15: 797–808. 18637074 10.1111/j.1365-2893.2008.01008.x

[32] Castelnuovo E, et al. (2006) The cost-effectiveness of testing for hepatitis C in former injecting drug users. Health Technology Assessment. [online]. 10: 32. 10.3310/hta10320 16948891

[33] Godin A, et al. (2021) The role of prison-based interventions for hepatitis C virus (HCV) micro-elimination among people who inject drugs in Montreal, Canada. International Journal of Drug Policy 88: 102738. 32278651 10.1016/j.drugpo.2020.102738

[34] Wong JB, et al. (2013) Cost-effectiveness of hepatitis c treatment by primary care providers supported by the Extension for Community Healthcare Outcomes (ECHO) Model. Hepatology. 64th Annual Meeting of the American Association for the Study of Liver Diseases: The Liver Meeting 2013. Washington, DC United States. 58: 330A.

[35] Manca F, et al. (2020) HCV screening strategies targeting prisoners and immigrants from endemic countries: are they cost-effective? Journal of Hepatology. EASL: The Digital International Liver Congress. 73: S806.

[36] Husereau D. et al. (2013) Consolidated Health Economic Evaluation Reporting Standards (CHEERS) – explanation and elaboration: a report of the ISPOR Health Economic Evaluation Publication Guidelines Good Reporting Practices Task Force. Value in Health: The Journal of the International Society for Pharmacoeconomics and Outcomes Research. 16: 231–250. 23538175 10.1016/j.jval.2013.02.002

[37] Evans D. (2003) Hierarchy of evidence: a framework for ranking evidence evaluating healthcare interventions. Journal of Clinical Nursing. 12: 77–84. 12519253 10.1046/j.1365-2702.2003.00662.x

[38] The SROI Network (2012) A guide to social return on investment. Available at: https://socialvalueuk.org/wp-content/uploads/2016/03/The%20Guide%20to%20Social%20Return%20on%20Investment%202015.pdf. (accessed 25 October 2023).

[39] Plugge E, et al. (2017) WEPHREN: a global prison health research network. International Journal of Prisoner Health. 13: 65–67. 28581374 10.1108/IJPH-03-2017-0014

[40] Pedrana A. et al. (2020) Global hepatitis C elimination: an investment framework. The Lancet. Gastroenterology & Hepatology. 5: 927–939. 32730786 10.1016/S2468-1253(20)30010-8

[41] Tricco AC, Lillie E, Zarin W, O’Brien KK, Colquhoun H, Levac et al. (2018) PRISMA extension for scoping reviews (PRISMAScR): Checklist and explanation. Annals of Internal Medicine 169: 467–473. 10.7326/M18-0850. 30178033

